# Dental root elevator embedded into a subgingival caries: a case report

**DOI:** 10.1186/s13104-015-1011-5

**Published:** 2015-02-28

**Authors:** Jaume Miranda-Rius, Lluís Brunet-Llobet, Eduard Lahor-Soler, Ombeni Mrina, Albert Ramírez-Rámiz

**Affiliations:** Departament d’Odontostomatologia, Facultat d’Odontologia, Universitat de Barcelona, Feixa Llarga s/n, 08907, L’Hospitalet de Llobregat, Barcelona, Spain; Servei d’Odontologia, Hospital Universitari Sant Joan de Déu - UB, Esplugues de Llobregat, Barcelona, Spain; Dental and Oral Department, Soweto General Hospital, Arusha, United Republic of Tanzania

**Keywords:** Tooth elevator, Instrument breakage, Radiological finding, Surgical stainless steel

## Abstract

**Background:**

Breakage of surgical instruments is a rare complication. A mistake in operator technique or sub-standard/aged tools could lead to this type of accident. A tooth elevator is an instrument used in minor oral surgical procedures to luxate the tooth or fractured root from its socket. The authors have not found any previously published cases reporting the breakage of a tooth elevator tip which then remained as a foreign body in a hidden caries cavity.

**Case presentation:**

A 28-year-old African black male was referred to a hospital in Tanzania for an intraoral radiography. The patient explained that six months previously his mandibular left third molar had been extracted. Whilst the healing process had been satisfactory, he had recently experienced acute oral pain in this region. The dental X-ray showed an image consistent with a piece of broken metal embedded in a distal subgingival caries at the mandibular left second molar.

**Conclusion:**

Oral and dental surgeons should take particular care when employing metal instruments with strong force in poorly visible areas. A radiographic study should be carried out when instrument breakage occurs. If an unexpected accident takes place during a surgical procedure, the patient must be informed in accordance with ethical codes, and suitable measures adopted to resolve the issue.

## Background

Foreign bodies in the oropharyngeal region are a problem frequently faced by otolaryngologists and dental surgeons. The accidental ingestion of sharp elements such as fish bones or toothpick pieces is quite common in the oral cavity and removal can be easily performed through simple manipulation. However, other foreign bodies in this region may require a surgical approach and/or endoscopic techniques under general anaesthesia [1,2].

Accidents can take place during surgery due to a number of factors including operator technique and sub-standard or aged instruments. Manufacture is strictly controlled, particularly in the case of dental, medical and surgical instruments which could cause serious injury to patients if they proved to be faulty. Occasionally, however, alterations in manufacturing technique or ineffective quality control occur and they are employed unknowingly [3].

**Figure 1:**
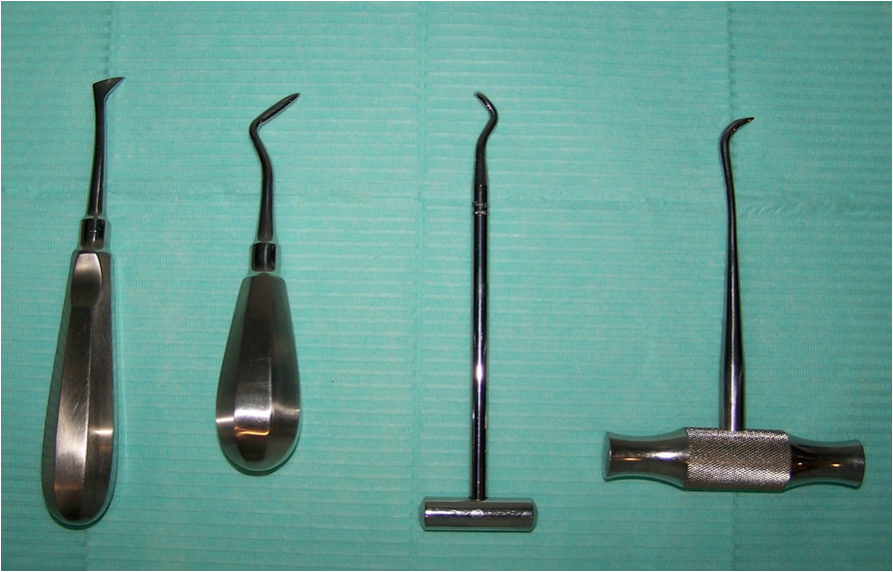
**Luxating dental elevators with curved bladed tips to facilitate root extraction.**

A tooth elevator is an instrument used in minor oral surgical procedures to luxate the tooth or fractured root from its socket. There is a vast range of elevator designs, including right and left sides for several types (Figure 1). Alveolar bone fracture and fracture/luxation of the adjacent tooth are two common problems associated with their use.

It is well known that any tooth extraction requires a preoperative radiograph to obtain information about root morphology and possible concomitant dental pathology such as interproximal caries or apical lesions. The aim of the present report is to describe an incidental radiographic finding that was consistent with a tooth elevator tip which had remained as a foreign body in a hidden caries cavity for approximately six months.

## Case presentation

**Figure 2:**
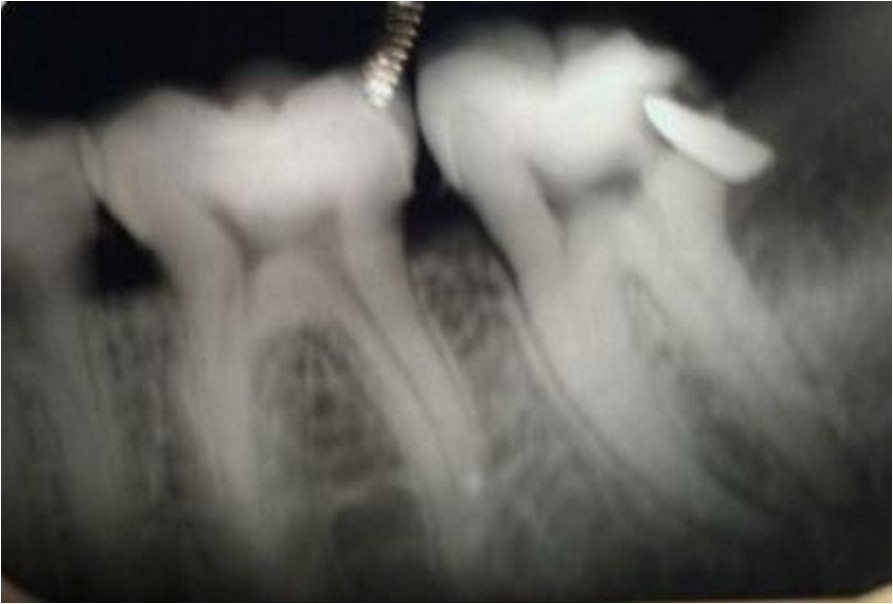
**Periapical radiographic image: Notice the curved blade elevator tip broken and embedded in a subgingival caries cavity of the mandibular left second molar (37).** Observe two radiolucent apical lesions in the affected molar (37) and a slight radiopacity located on the distal part of the first molar crown (36), which was a consequence of the Kocher clamp used during a manual developing process.

A 28-year-old African black male was referred to a hospital in Tanzania for an intraoral radiography. His major complaint was an acute, diffuse-patterned oral pain in the left mandibular area. The patient had no significant previous medical history. A clinical examination confirmed satisfactory dental hygiene without any apparently relevant findings. The patient explained that his mandibular left third molar had been extracted six month previously and that the surgery had been quite laborious and lengthy. Although the healing process had been satisfactory, considerable pain had recently begun in this area. The dental X-ray showed an image consistent with a piece of metal embedded in a subgingival distal caries at the mandibular left second molar (37), which was probably related to a previous tooth impaction process of the mandibular left third molar (38). Two periapical lesions detected at the mandibular left second molar roots might have justified his acute tooth ache due to acute exacerbation (Figure 2).

Initially, the differential diagnosis of this radiographic finding was that of a possibly loosened or released piece of amalgam. Nevertheless, the patient had never previously undergone any dental fillings. After repeating the X-ray twice in order to rule out a possible image artefact, it was concluded that during the third molar extraction a curved blade elevator tip had fractured and impacted into the subgingival caries cavity. As the patient had only been referred for a dental X-ray he was given a written note explaining the finding. His dentist was informed about the need to perform an occlusal access cavity in order to initiate a root canal treatment and remove the foreign body from the second molar (37).

### Discussion

The breakage of some instruments, such as endodontic files and dental burs, due to a number of factors including defective manufacturing, stress, fatigue, rust, and poor handling is not unknown in dentistry [4]. Few papers, however, in the literature have dealt with the breakage of instruments used for exodontias. Yasuhara *et al.*, registered various medical accidents caused by defective surgical instruments over two years. In the maxillofacial speciality the authors reported 7 incidences out of 548 operations [5]. According to Kluess *et al*., it is important that any incident with orthopaedic surgical instruments should be reported to the manufacturer and the health authorities for sufficient processing and risk assessment of the accident [6]. In the case of some reusable metal instruments both titanium alloys and stainless steel are in the high performance range. The latter is the most widely used material for instruments and, according to surgical requirements, its alloys vary: the most frequent being martensitic and austenitic stainless steels. Biomedical cutting instruments are often made of martensitic stainless steel due to its pronounced durability coupled with acceptable corrosion resistance. Surgical instruments that may be subject to high pressure forces, such as a dental elevator, are composed of austenitic stainless steel as it is less brittle [7]. A safe and effective elevator should have extreme values for torque, and high stress values [8].

Surgical instruments manufactures should carry out strict quality controls and have their instruments bear a visible mark as a sign of guarantee. Various authors have suggested that the inferior quality of some surgical instruments may be a reflection of poor working conditions and low standards, particularly in the developing world. Responsibility lies with the suppliers from developed countries manufacturing in the developing world who behave in an unethical manner, maximising profits and minimising the remuneration of the people who actually produce the goods [9-11].

The location and retrieval of broken fragments during a tooth extraction procedure should not be a serious problem, in most cases the fragment is immediately identified. Any instrument breakage implies the obligation to search for the fractured fragment and remove it in order to avoid possible infection and prevent complications due to swallowing or aspiration of the fragment [3]. Some of the metallic pieces of the surgical instrument could end up in a fibrous issue capsule and gain access to the adjacent spaces [12]. The case we report here is unusual in that the elevator tip was embedded in a subgingival caries and remained there asymptomatically for approximately six months.

At present there are a number of radiological explorations to identify metallic foreign objects. Cone beam computerized tomography (CBCT) is an excellent tool to locate metallic foreign objects [13]. However, a single periapical radiograph or using more than one radiograph to apply a tube-shift technique, may be sufficient. Whenever possible, simpler techniques should be first applied. In addition, occlusal radiography can also be employed as necessary. If an occlusal film is not available a periapical radiograph can be put on the surface of the tooth, or on the edentulous crest, and this may reveal an embedded foreign object. If with using these techniques, the foreign object is not detected, then other more sophisticated techniques such as CBCT should be applied. In our case, only conventional intraoral periapical radiographic imaging was available, due to limited hospital facilities, to inform the referral doctor about our findings.

The care of medical and surgical instruments has a decisive effect on their efficiency and durability. Damage due to breakage and scratches is usually caused by their being incorrectly stored and secured during sterilization, or being carelessly positioned in the treatment area thus favouring falls. Additionally, metal instruments used in clinical practice may be subjected to fatigue due to the effects of sterilization processes. Manufacturers recommend that all instruments made of metal should be checked regularly before packaging in order to diminish the risk of possible incidences [14].

The concept of honest and correct communication between physician and patient is a crucial issue. It is linked to the disclosure of adverse events and errors, a complex topic covering medical, psychological, legal, and ethical aspects [15-17]. Current socio-cultural trends, which condition the medical profession in a variety of ways, and a greater focus on the dignity and rights of the patient, have led to a growing tendency to fully inform patients with regard to their illness, progress, and therapy. Nevertheless, it is still common for doctors to choose to remain silent or manipulate the truth in some way, especially when the prognosis or accident is serious or negative and the patient seems to be having real difficulties accepting it [18]. As clinicians, this reported case should be helpful by reminding us of the importance of our ethical code with respect to the patients, especially when an adverse eventuality appears during a surgical procedure.

## Conclusion

In dentistry is always advisable to use good quality and reliable brands for any instrument. Whenever any retention of a broken metal instrument is suspected an imaging radiological study will indicate its position and help avoid potential surgical complications. Preoperative and postoperative check-ups of instruments are also essential. Dental and oral surgeons should be particularly careful when metal instruments deployed with strong force are used in poorly visible areas such as the third molar region. If an unexpected accident takes place during a surgical procedure the patient should be informed in accordance with ethical codes, and suitable measures adopted to resolve the issue.

## Consent

Written informed consent was obtained from the patient for publication of this Case Report and any accompanying images. A copy of the written consent is available for review by the Editor-in-Chief of this journal.

## Abbreviation

CBCT: Cone beam computerized tomography
